# Supplementary figures and images for: Discovery and validation of molecular patterns and immune characteristics in the peripheral blood of ischemic stroke patients
Source: PeerJ. 2024 Apr 19;12:e17208. doi: 10.7717/peerj.17208 (PMC11034498; doi:10.7717/peerj.17208)

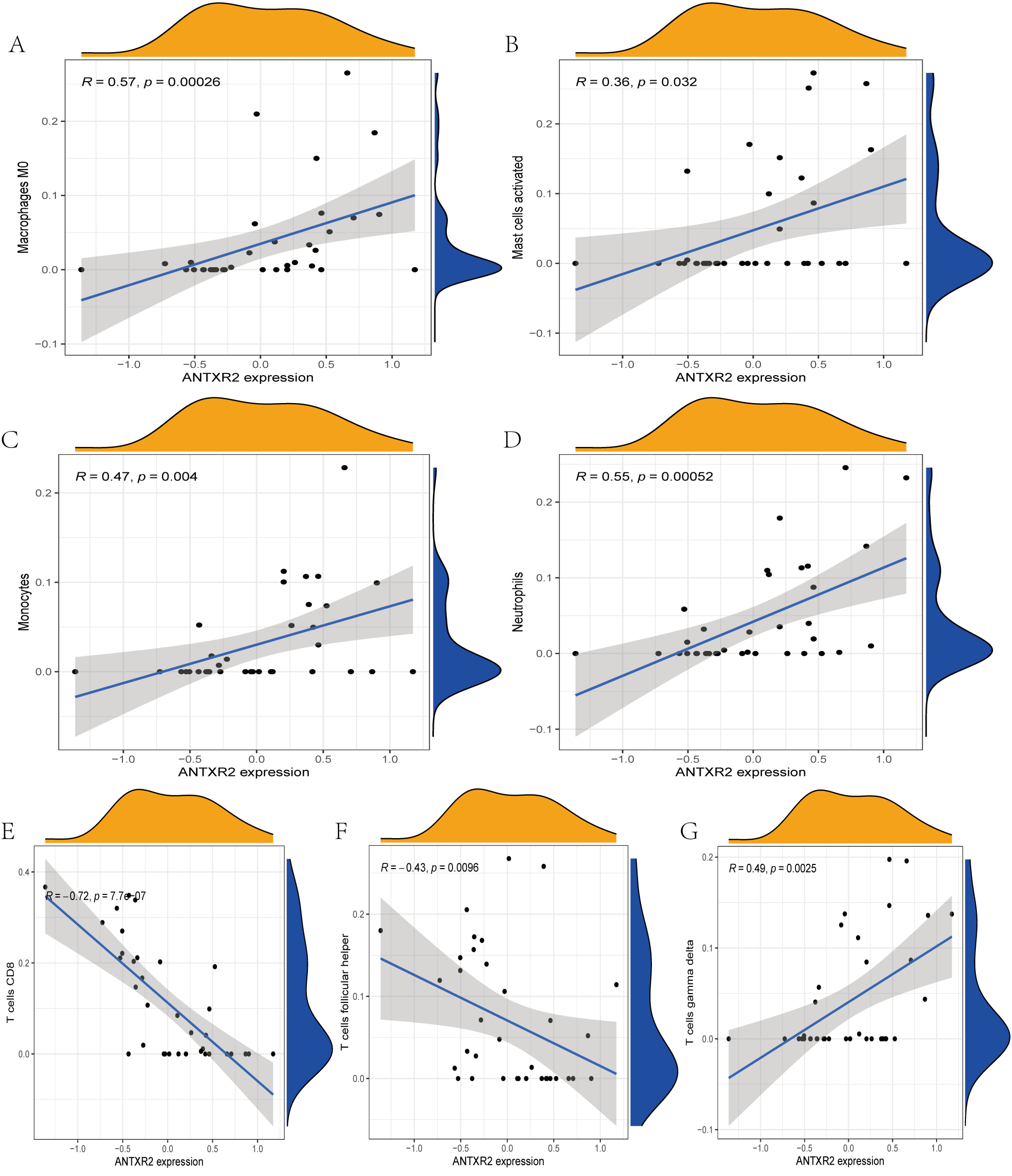

Supplement: Supplemental Information 1 [file peerj-12-17208-s001.png]

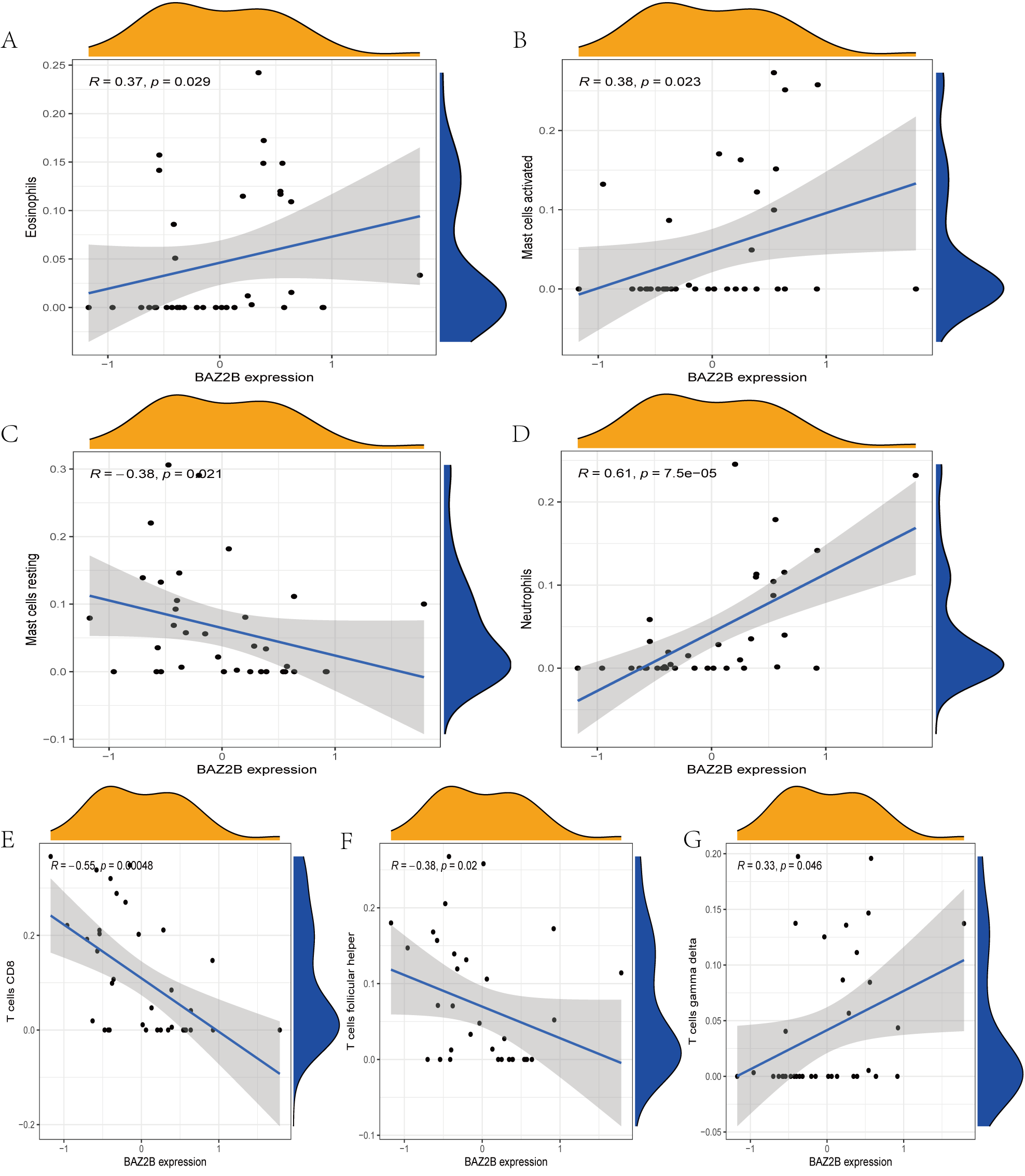

Supplement: Supplemental Information 2 [file peerj-12-17208-s002.png]

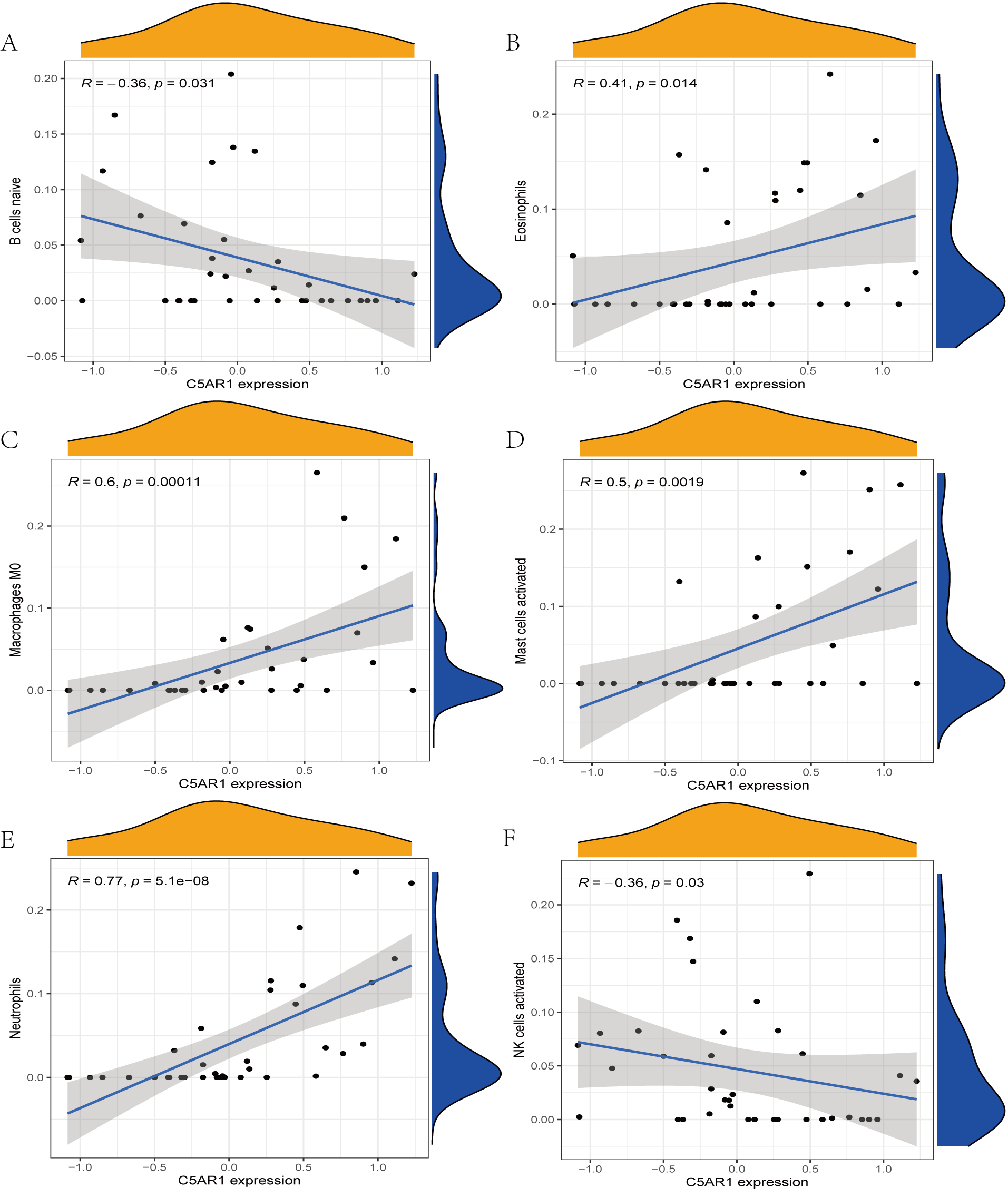

Supplement: Supplemental Information 3 [file peerj-12-17208-s003.png]

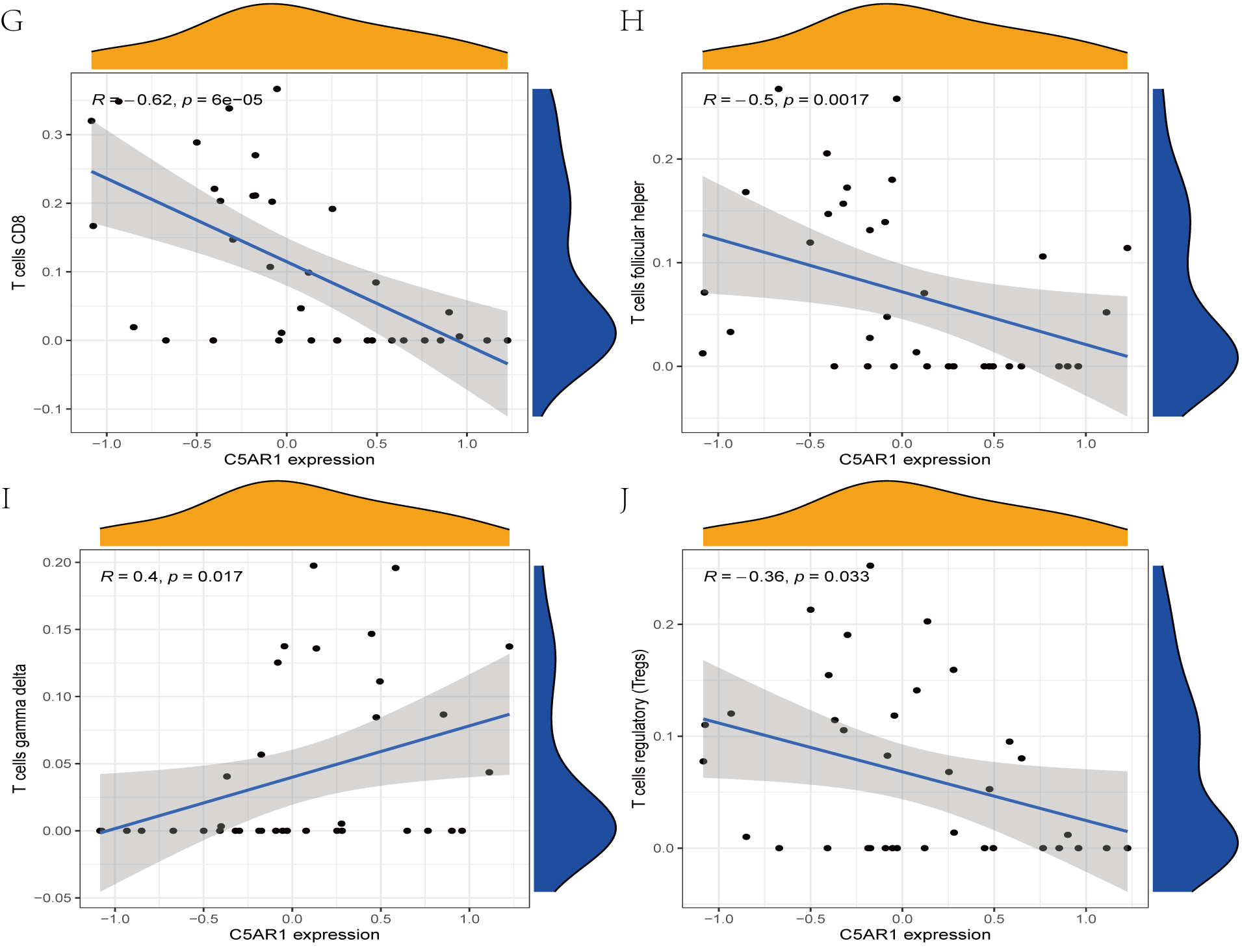

Supplement: Supplemental Information 4 [file peerj-12-17208-s004.png]

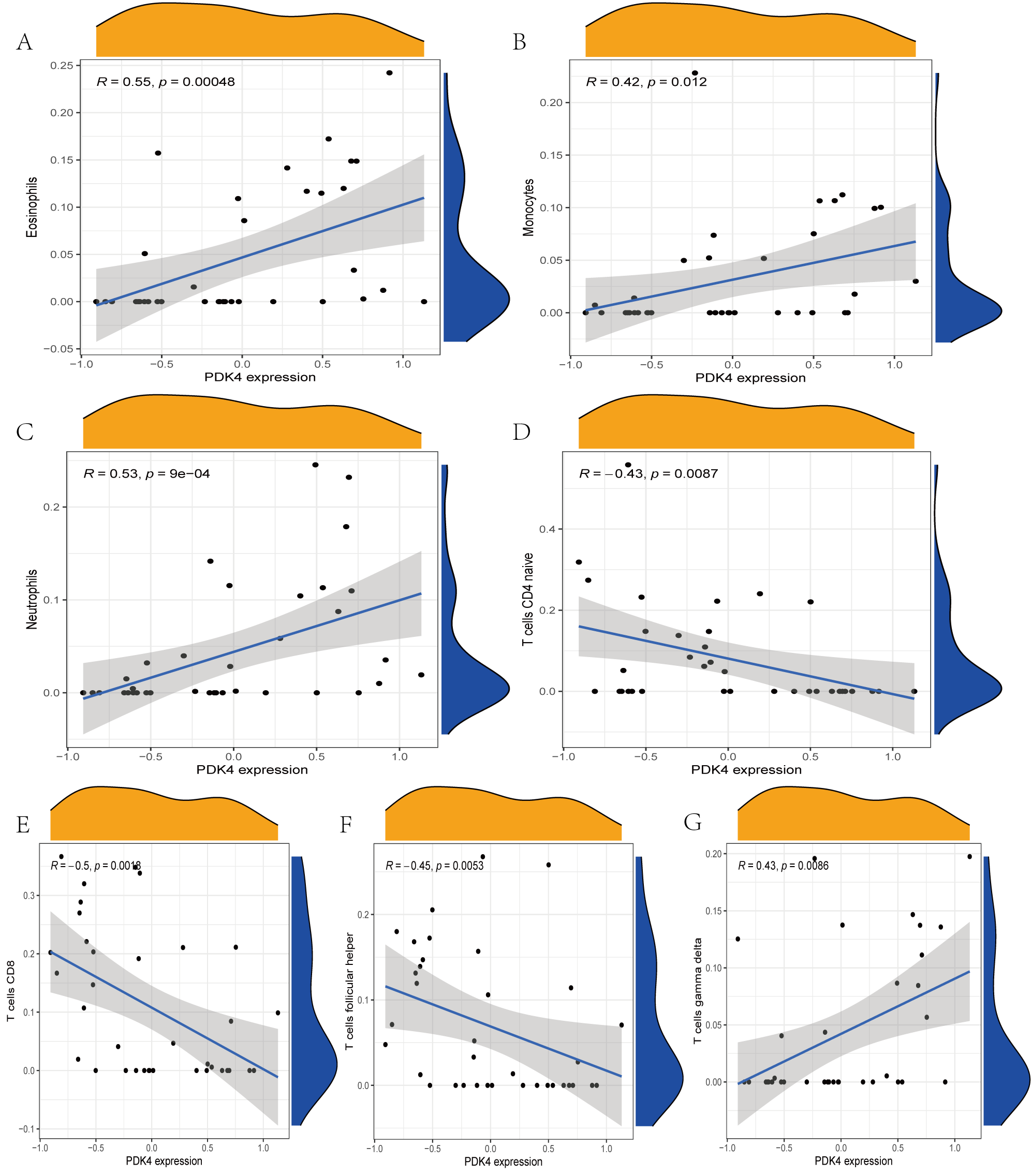

Supplement: Supplemental Information 5 [file peerj-12-17208-s005.png]

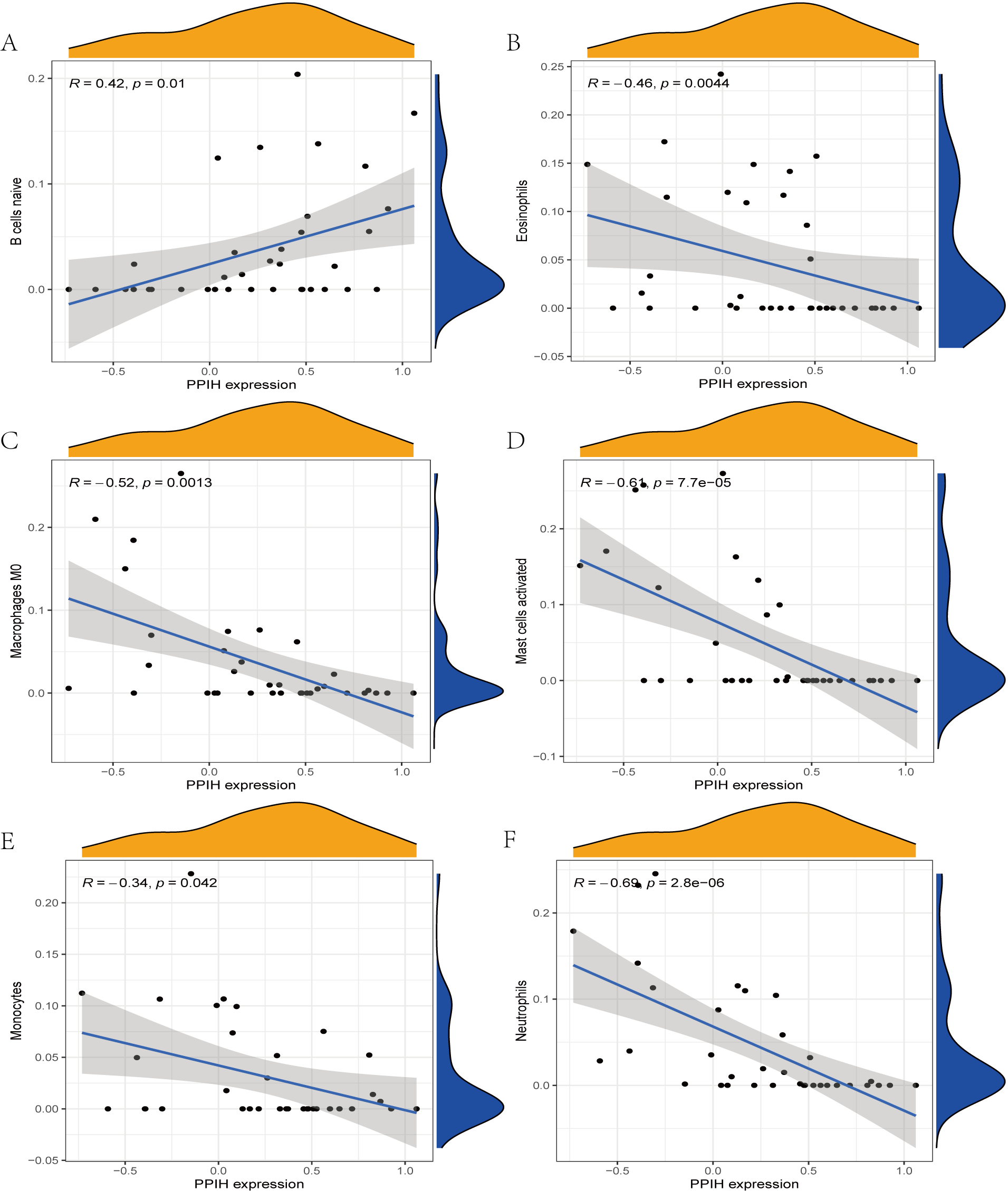

Supplement: Supplemental Information 6 [file peerj-12-17208-s006.png]

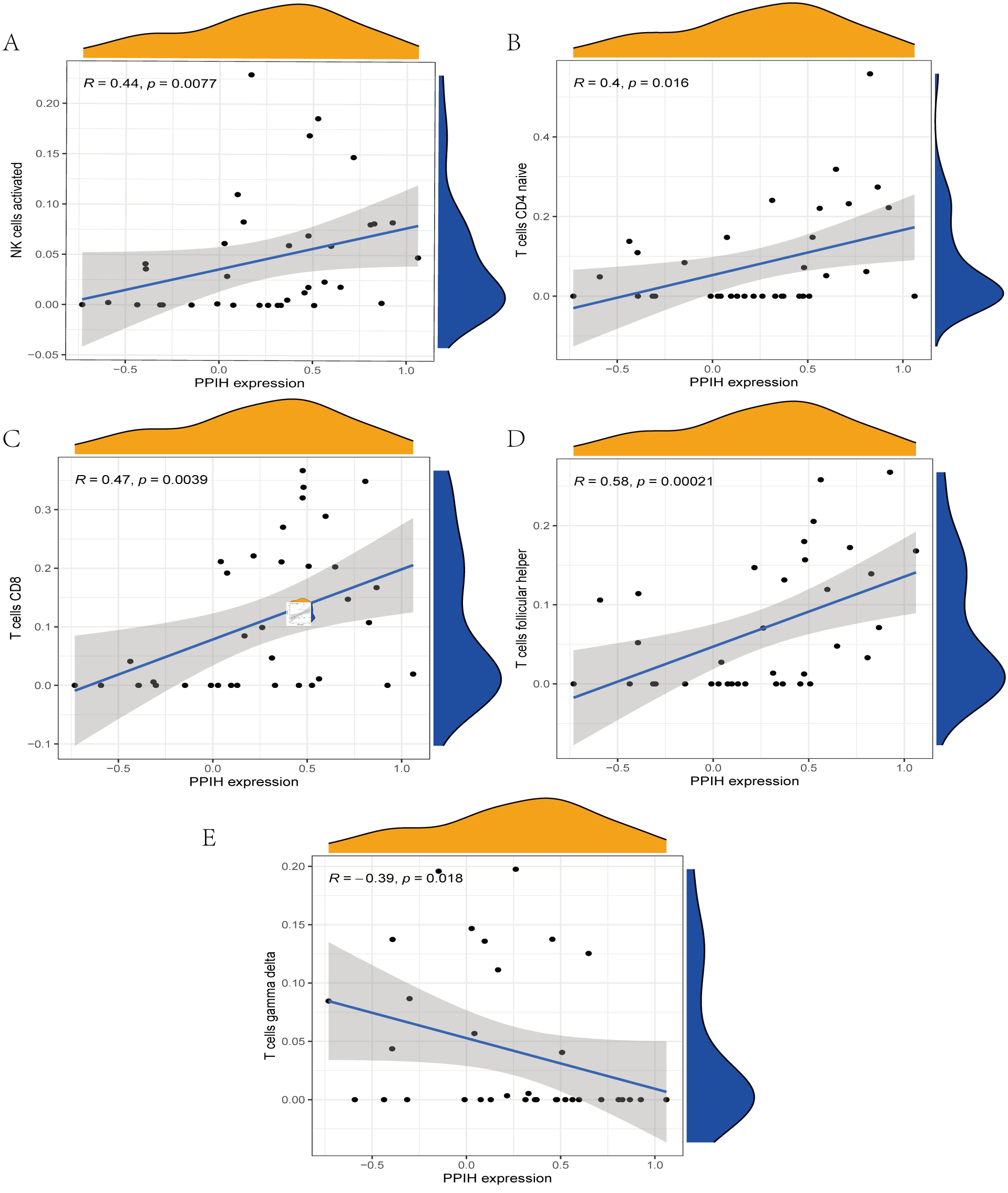

Supplement: Supplemental Information 7 [file peerj-12-17208-s007.png]

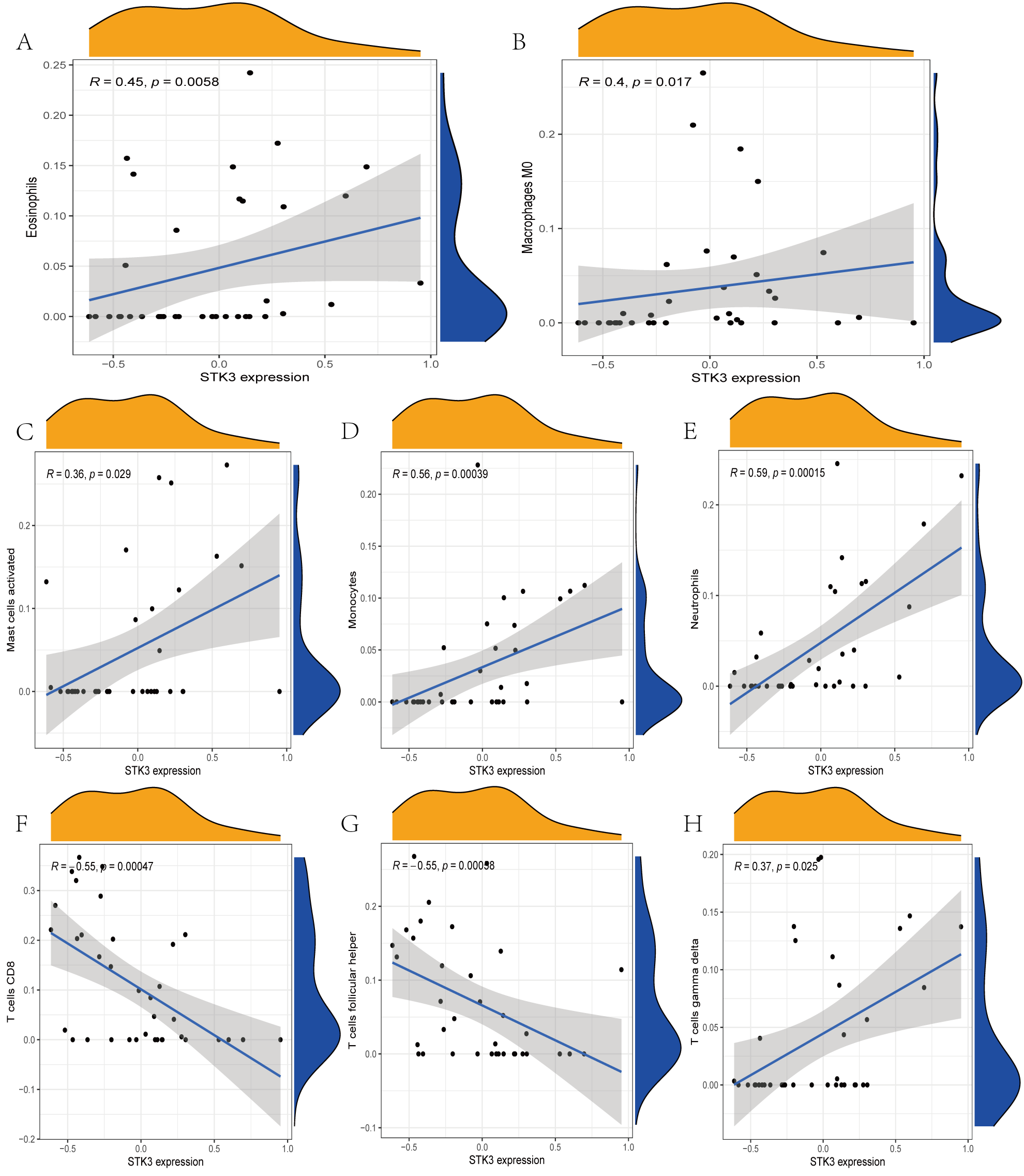

Supplement: Supplemental Information 8 [file peerj-12-17208-s008.png]
