# Supplementary material for: Discovery and validation of molecular patterns and immune characteristics in the peripheral blood of ischemic stroke patients
Source: PeerJ. 2024 Apr 19;12:e17208. doi: 10.7717/peerj.17208 (PMC11034498; doi:10.7717/peerj.17208)
Supplement: Supplemental Information 9 [file peerj-12-17208-s009.docx]

S3 Primer information

| **Gene** | **Primer name** | **Primer (5’——>3’)** |
| --- | --- | --- |
| ANTXR2 | CL-ANTXR2-F | CTTCCTATTATGGTGGTCG |
|  | CL-ANTXR2-R | TGATGGGTTCCTCTGTTT |
| BAZ2B | CL-BAZ2B-F | CTCGTAACCTCCACATAG |
|  | CL-BAZ2B-R | TATTACTTCAGGGTACTGC |
| C5AR1 | CL-C5AR1-F | CGCTTTCTGCTGGTGTTT |
|  | CL-C5AR1-R | TTTGTCGTGGCTGTAGTCC |
| PKD4 | CL-PDK4-F | CAGGAAACCCAAGCCACA |
|  | CL-PDK4-R | TTCCCAAGACAACAATAACCTC |
| PPIH | CL-PPIH-F | TGATTCAGGGTGGAGATT |
|  | CL-PPIH-R | ACTTAGAGCAGGTGATAAAGA |
| STK3 | CL-STK3-F | ATGGCTCCTGAGGTGATT |
|  | CL-STK3-R | GTTGGTGGTGGATTTGTG |
